# Evaluating the validity of an integrity-based situational judgement test for medical school admissions

**DOI:** 10.1186/s12909-015-0424-0

**Published:** 2015-09-02

**Authors:** Adrian Husbands, Mark J. Rodgerson, Jon Dowell, Fiona Patterson

**Affiliations:** 1 Assessment Lead and Senior Lecturer, School of Medicine, University of Buckingham, Hunter Street, Buckingham, MK18 1EG UK; 2 Fourth Year Medical Student, School of Medicine, University of Dundee, Dundee, UK; 3 Admissions Convenor and Reader in Medical Education, School of Medicine, University of Dundee, Dundee, UK; 4 Work Psychology Group, 27 Brunel Parkway, Pride Park, Derby, DE24 8HR UK

## Abstract

**Background:**

While the construct of integrity has emerged as a front-runner amongst the desirable attributes to select for in medical school admissions, it is less clear how best to assess this characteristic. A potential solution lies in the use of Situational Judgement Tests (SJTs) which have gained popularity due to robust psychometric evidence and potential for large-scale administration. This study aims to explore the psychometric properties of an SJT designed to measure the construct of integrity.

**Methods:**

Ten SJT scenarios, each with five response stems were developed from critical incident interviews with academic and clinical staff. 200 of 520 (38.5 %) Multiple Mini Interview candidates at Dundee Medical School participated in the study during the 2012–2013 admissions cycle. Participants were asked to rate the appropriateness of each SJT response on a 4-point likert scale as well as complete the HEXACO personality inventory and a face validity questionnaire. Pearson’s correlations and descriptive statistics were used to examine the associations between SJT score, HEXACO personality traits, pre-admissions measures namely academic and United Kingdom Clinical Aptitude Test (UKCAT) scores, as well as acceptability.

**Results:**

Cronbach’s alpha reliability for the SJT was .64. Statistically significant correlations ranging from .16 to .36 (.22 to .53 disattenuated) were observed between SJT score and the honesty-humility (integrity), conscientiousness, extraversion and agreeableness  dimensions of the HEXACO inventory. A significant correlation of .32 (.47 disattenuated) was observed between SJT and MMI scores and no significant relationship with the UKCAT. Participant reactions to the SJTs were generally positive.

**Conclusions:**

Initial findings are encouraging regarding the psychometric robustness of an integrity-based SJT for medical student selection, with significant associations found between the SJTs, integrity, other desirable personality traits and the MMI. The SJTs showed little or no redundancy with cognitive ability. Results suggest that carefully-designed SJTs may augment more costly MMIs.

## Background

Medicine is massively oversubscribed and there is continued pressure on medical schools to select the best candidates from an ever-growing applicant pool [1–3]. With documented success on using assessments of cognitive skills such as academic criteria and aptitude tests [4, 5], the focus has shifted to selection based on desirable personal characteristics. With over 87 different personal characteristics identified, and no general consensus in the literature [6], further careful attention needs to be paid to how to best select for which characteristics.

From the emerging work on identifying important personal attributes, the construct of integrity has emerged as a clear front-runner [7, 8]. Koenig et al. [7] attempt to address this lack of consensus in a large-scale multi-centre study which involved 98 North American medical schools to identify core personal characteristics and included literature reviews, role analyses and questionnaires. These authors identified ‘ethical responsibility to self and others’, followed by social skills as the most important among 9 personal competencies for successful medical school entrants. Another large-scale study by Patterson et al. [8] synthesized findings from a literature review, semi-structured interviews, surveys and an expert panel involving 122 faculty from medical and dental schools across the UK, and found that communication followed by ‘integrity’ was seen as most important to evaluate at selection. The Patterson et al [8] operationalization of integrity largely subsumes the Koenig et al. [7] definition of ‘ethical responsibility to self and others’ and included “honesty to self and others”, “willingness to hold others to account”, “maintaining confidentiality” and “willingness to challenge unacceptable behaviour”. With the construct of integrity being assigned such prominence, establishing a suitable vehicle to assess it reliably is highly desirable.

Whilst there has been some attempt to measure integrity-related constructs in medical school admissions using the Multiple Mini Interview (MMI), this is a sub-optimal solution for a number of reasons. Firstly MMIs typically focus on measuring communication skills relative to all other constructs. The two MMIs with demonstrable predictive validity in the literature focus on communication skills, with five out of ten and two out of ten stations measuring integrity-related constructs as described by MMI researchers at Dundee [9, 10] and McMaster Universities [11, 12] respectively. Eva et al. [11] admit that while MMI developers may attempt to measure integrity-related constructs, ratings assigned by MMI assessors are likely to be influenced by candidates’ ability to communicate effectively. This may result in somewhat ‘contaminated’ integrity scores where integrity-related MMI ratings are likely to be heavily confounded with interpersonal skills.

MMIs also present practical constraints, as it is often not feasible to interview all applicants because of the significant costs incurred by both them and institutions [10]. This exposes a weakness in pre-interview selection systems which rarely include any robust non-cognitive assessment, and none assessing integrity specifically [7].

A viable solution to the assessment of integrity may lie in the use of Situational Judgement Tests (SJTs), where test-takers are presented with job-related scenarios and asked to indicate their response(s) according to predetermined options [13, 14]. In addition to strong psychometric evidence [15–17], the SJT format has gained popularity in advanced-level high-stakes selection because their machine-scorable format allows them to be administered to a large number of applicants before the interview process, unlike high-fidelity simulations such as assessment centre exercises and MMIs.

A handful of authors have investigated SJTs for undergraduate medical school selection. In the only study pertaining to graduate entry selection Dore et al. [18] administered an SJT to a total of 277 participants in two separate studies which aimed to measure a range of constructs including collaboration, communication, professionalism and confidentiality. While the authors found acceptable reliability and statistically significant correlations with MMI score (.51) the resource intensive nature of marking their short-answer (free text) response format make results less generalisable and potentially discourage widespread adoption.

Lievens and colleagues assessed SJTs among school-leaving (post-secondary education) prospective medical and dental students in a number of studies, demonstrating strong psychometric properties [19–22]. Lievens and Coetsier [19] examined the construct and predictive validity of an SJT undertaken by 941 medical and dental students, and found that SJT scores correlated significantly with openness personality dimensions (*r* = .10 to .15) and first year examination scores (*r* = .23), with incremental validity over cognitive ability of 3.1 %. Lievens, Buyse and Sackett [20] continued to examine the psychometric properties of the SJT in a longitudinal study of 7197 students across the first four years of undergraduate studies and found significant correlations ranging from .10 to .38 across Grade Point Averages (GPAs) on interpersonal skills components, becoming more valid through the years. A further study by Lievens and Sackett [21] found that a video-SJT significantly predicted interpersonal skills GPA among 145 first year students (*r* = .35). The most recent study to date by Lievens [22] utilised a longitudinal, multiple cohort analysis of 5444 medical school candidates and revealed significant correlations ranging from .08 to .21 between SJT scores and various undergraduate GPA and postgraduate outcome measures, including supervisory ratings of job performance (*r* = .15) and a final General Practice (GP) postgraduate OSCE (*r* = .12). Despite these encouraging results, it must be noted that analyses in all but the final study investigated both medical and dental students, making conclusions for medical students as a specific group difficult to infer. Furthermore, SJTs in the aforementioned studies were all constructed to measure interpersonal skills with the exception of the first study where the construct being measured was not made explicit. There is therefore still a need to investigate whether a constructs such as integrity can be successfully developed to be administered on a large scale.

The few studies which investigate the psychometric properties of an integrity-related SJT have demonstrated good construct and predictive validity. De Meijer et al. [23] observed a small correlation of .23 between with a video-based SJT for 203 Dutch Police Officers and an integrity-related personality dimension of the HEXACO personality scale. Becker [24] also showed that scores on an integrity-related SJT correlated with managers’ ratings of workplace performance among 273 retail employees through evaluation of their workplace performance in terms integrity related outcomes, with validity coefficients ranging from 0.18 to 0.26 [24, 25]. As these studies have been conducted in an occupational setting, further research is needed to investigate whether similar principles can be applied to assessing and selecting for integrity among prospective medical students.

Admissions selectors wishing to investigate SJTs should be aware of concerns by a number of authors that a proportion of the variance of SJTs scores may be explained by cognitive ability [17, 26–28]. In the context of medical student selection, this would be undesirable for two reasons. Firstly, a relationship between SJT performance and cognitive ability may be suggestive of redundancy of the assessment tool. Secondly, cognitive ability tests are often associated with adverse impact against certain ethnic minority subgroups [8, 17, 28–30], an effect that test developers would clearly wish to avoid to ensure fairness.

The present study aims to explore the psychometric properties of an SJT designed to measure the construct of integrity by examining the associations between SJT score, personality, cognitive ability, other pre-admissions measures, and acceptability among participants. This is important to determine if an SJT could usefully screen for desirable core personal competencies which could add value to, or improve efficiency of, the selection process. To our knowledge, this study is unique as it represents the first attempt to assess integrity using a specifically - focused SJT in a medical school selection setting.

## Methods

### Procedure

Data collection took place at Dundee Medical School in conjunction with the MMI process in December 2012 and January 2013. One thousand, six hundred and ninety-five applicants applied to Dundee and met the minimum requirements, out of which 520 (30.7 %) attended for interview based on their academic grades and UKCAT score. Two hundred (38.5 %) MMI candidates agreed to participate in the study. MMI candidates were invited to participate after completion of their interview, before they were aware of any selection decisions and were informed that participation would not affect the selection decisions in any way. All participants were asked to complete the SJT in written booklets along with the HEXACO-PI and an acceptability questionnaire. No incentives were offered to participants. Scores from study measures were matched to those of admissions tools, namely MMI and UKCAT scores. Ethical approval for this study was obtained from the University of Dundee Ethics Committee (UREC 12155).

### Study measures

#### Situational judgement test (SJT)

The construct of integrity was operationalized based on a review of the literature identifying integrity-related constructs including: honesty; adherence to a moral or ethical code; opposition to acts of fabrication, plagiarism, cheating and dishonesty; a strong sense of justice and fairness; knowledge of confidentiality and sincerity [31–33]. The SJT consisted of 50 scorable items pertaining to ten scenarios involving medical student integrity, each elicited through interviews conducted with 12 members of faculty using the critical incident technique [34]. All interviewees were involved in the delivery of professionalism teaching in the undergraduate curriculum. Items were further developed and piloted with separate groups of senior and junior medical students, and further reviewed by a senior physician. Scenarios were presented to roughly equal groups of participants in one of three equivalent formats: a paragraph of written prose, a short video or a verbatim transcript of the video. The scenario content and scoring key applied to all formats were identical and as results were broadly similar across formats, the combined SJT scores were the focus of this study. Table 1 summarises the SJT scenario content.

**Table 1:** SJT scenario content

Scenario	A medical student…
1	reveals to another that he was not genuinely ill even though he phoned in sick to a tutorial session
2	is questioned about extreme and provocative comments on her Facebook page
3	overhears two fellow students discussing details of a patient’s condition in a café
4	approaches another student asking for the wording of a reference to be changed
5	is worried that her essay has been plagiarised by a friend
6	reveals she was told which topics not to revise for by someone who sat the same exam on a previous day
7	reveals he borrowed a book from a tutor but decided not to return it
8	observes a consultant speaking extremely condescendingly to a nurse
9	refuses to share notes with a fellow student who fails to contribute to group work
10	reveals he will only be attending an optional lecture for the free food provided

The SJT response items employed four-point likert scales [35] on which candidates assigned responses to each of the 50 items ranging from one (most appropriate) to four (least appropriate) with an even number of points used to deter respondents from using a neutral middle option throughout the assessment [17, 21, 28, 29].

For the purposes of scoring, 13 members of staff from the University’s Undergraduate Centre for Medical Education were asked to complete the SJTs as subject matter experts (SMEs). The Chan and Schmitt method was used to create a scoring key where participants were awarded either two, one or zero marks depending on whether their response matched that of more than fifty percent, more than twenty five percent, or less than twenty five percent of a pool of SMEs respectively [36, 37].

#### HEXACO personality inventory (HEXACO-PI)

To investigate the SJT’s construct validity the associations between SJT score and dimensions of the HEXACO personality inventory were determined. The HEXACO model consists of 6 dimensions, namely Honesty–Humility (H-H – High scores relate to honesty, sincerity), Emotionality (E- high scores relate to being emotional and anxious), eXtraversion (X – high scores relate to being outgoing and sociable), Agreeableness (A – high scores relate to being patient and gentle), Conscientiousness (C – high scores relate to diligence and thoughtfulness), and Openness to Experience (O – high scores relate to being intellectual and innovative) [38–41]. The HEXACO model is supported by lexical analysis of data collected from multiple existing studies [39], and has been translated into over 16 languages to date [41].

The authors were particularly interested in the relationship between SJT score and Honesty-Humility (H-H), a dimension which has been shown to be analogous to integrity, with demonstrable relationships between H-H and both overt and covert integrity tests [39, 40]. Low scores on the H-H dimension have been associated with workplace deviance and unethical business practices among public and private-sector employees, workplace delinquency within university students with an employment history, and manipulation, exploitation and Machiavellianism among undergraduate students [38]. Within the higher education student population, the H-H dimension has also been shown to be an important predictor of academic performance both in terms of attainment scores as well as incidences of academic dishonesty [42].

#### Acceptability questionnaire

Acceptability was assessed using an adaption of the Smither et al [43] face validity questionnaire [20, 21, 44] and consisted of five likert-type questions. Questions (Table 2) included those related to job-relatedness (e.g. “I understood what the test had to do with the role of medical student”) and perceived fairness (e.g. “There was a real connection between the test and the role of medical student”). Scale points range from by 1 (strongly disagree) to 5 (strongly agree).

**Table 2:** Descriptive statistics for participants’ acceptability ratings

Item		Response frequency (%)					M^b^	SD^c^
		1 (SD)^a^	2 (D)	3 (N)	4 (A)	5 (SA)		
1	I understood what the test had to do with the role of medical student	0.0	3.5	3.0	60.5	31.0	4.2	0.7
2	I could see the relationship between the test and what is required of a medical student	0.0	2.0	1.5	51.5	43.0	4.4	0.7
3	It would be obvious to anyone that the test is related to the role of medical student.	4.0	18.5	22.0	40.0	13.5	3.4	1.1
4	The actual content of the test was clearly similar to those potentially encountered by medical students	1.0	6.0	18.5	50.0	22.5	3.9	0.9
5	There was a real connection between the test and the role of medical student	1.0	1.5	6.0	46.0	43.5	4.3	0.8

^a^*SD* Strongly Disagree, *D* Disagree, *N* Neither Agree nor Disagree, *A* Agree, *SA* Strongly Agree

^b^*M* Mean, ^c^*SD* Standard Deviation

### Admissions tools

#### Multiple-mini interview (MMI)

Dundee’s MMI consisted of 10 seven-minute interviews which involved a series of one-to-one interviews, interactive tasks and role play scenarios [9]. Interview content was developed based on a predefined set of desirable personal qualities determined by the medical school’s admissions committee. These were communication (including empathy), critical thinking, teamwork, motivation as well as moral reasoning and integrity.

The Dundee MMIs have been shown to be reliable and predictive of future performance in medical school [9, 10], which is consistent with emerging data supporting the use of MMIs due to robust psychometric properties [11, 44–48]. Further details on the development of the Dundee MMIs are provided in Dowell et al. [9].

#### United Kingdom Cognitive Ability Test (UKCAT)

The UKCAT is an intelligence (or aptitude) test designed to "assess a range of mental abilities identified by University medical and dental schools as important" [49]. The UKCAT is distinct relative to other well-known cognitive ability tests such as the Medical College Admissions Test (MCAT) and BioMedical Admissions Test (BMAT) because it aims to purely assess aptitude and contains no knowledge-based component. The assessment consists of four subtests: a quantitative reasoning test, decision analysis assessment, a verbal reasoning test, and an abstract reasoning exercise. For the purposes of this study the total UKCAT score was used in the analyses.

#### Academic score

Numerical scores were assigned to applicants’ academic qualifications on a six-point scale according to their level of achievement obtained from their Universities and Colleges Admissions Service (UCAS) form. Qualifications included A-levels, Scottish Highers (equivalent to A-levels in Scotland), degree classifications and other relevant qualifications.

#### Analysis

Data were analysed with SPSS 21.0 for Windows (SPSS, Inc., Chicago, IL, USA). Histograms and plots were used to check for unexpected and outlying values and to assess the data for normality. Independent variables were academic, MMI, UKCAT, SJT and HEXACO scores and acceptability ratings. Applicants with missing data for a particular variable were omitted from the statistical analyses involving that variable.

To determine the underlying patterns among SJT scores, Exploratory Factor Analysis (EFA) by principal components with varimax rotation was conducted. This statistical method analyses the correlations between assessment scores with the aim of revealing whether groups of theoretically similar scores correlate [50].

Pearson’s correlation coefficient was used to measure associations between SJT score and other independent variables. Interpretation of correlations was undertaken using Cohen’s correlation effect size descriptors [25]. Analysis of the acceptability questionnaire was undertaken using descriptive statistics.

## Results

Scores from two participants who failed to complete a large proportion of SJT and HEXACO items were discarded. Final analysis pertained to 198 participants, all of whom completed the MMI, SJT, HEXACO inventory and acceptability questionnaire.

Eighty-seven (43.9 %) participants were male and 111 (56.1 %) female. A chi squared test revealed no statistically significant differences between SJT participants and MMI candidates by gender, χ^2^ = .89, *p* = .93. The average age among participants was 18 years and 4 months. There were no significant differences between the ages of SJT and MMI candidates, *t*(518) = 1.51, *p* = .25.

There were no statistically significant differences between mean scores of SJT participants on the MMI as compared to all interviewed candidates; *t* (518) =1.24, *p* = .21. UKCAT scores were not available for five applicants due to deferred entry, prohibitive medical condition or geographic location preventing them from attending a UKCAT testing centre. There were no significant differences between the mean UKCAT scores of SJT participants and all MMI candidates, UKCAT: *t* (513) = .72, *p* = .46. Academic scores were not available for five applicants who applied under a widening access initiative. Academic scores for SJT candidates were significantly lower (Mean = 5.0, SD = 0.5) than those of all MMI participants (Mean = 5.1, SD = 0.6), *t*(513) = 2.31, *p* < .05. A small Cohen’s d [25] effect size of 0.18 was associated with this comparison.

In keeping with best-practice, 14 SJT items were found to contribute negatively to the Cronbach’s alpha and were removed from the final analysis [51]. The Cronbach’s alpha of the remaining items was 0.64. Female participants (*M* = 35.0, SD = 7.4) scored significantly higher than males (*M* = 31.7, SD = 7.0) on the SJTs, *t*(196) = 3.21, *p* < .01. A medium Cohen’s d effect size of 0.46 was associated with this comparison.

Table 3 shows item-level statistics for the 36 items included in the final analysis, with means, maximum scores and difficulty (%) of each item according to scenario. The average difficulty rating was 51.3 % and ratings ranged from 16.0 to 87.3 %.

**Table 3:** Item-level statistics for SJT items

Scenario	Item	Maximum score	Mean score	Difficulty (%)
1	1	2	1.34	67.0
	2	2	.39	19.5
	5	2	1.43	71.5
2	1	2	.74	37.0
	3	2	1.44	72.0
	4	1	.72	72.0
	5	2	.93	46.5
3	2	2	1.26	62.8
	3	1	.75	75.0
	4	2	1.20	60.0
4	1	1	.51	50.5
	2	2	.72	35.8
	3	2	.37	18.5
	4	2	1.43	71.5
	5	2	1.72	86.0
5	1	2	.70	35.0
	4	2	.40	20.0
	5	2	1.75	87.3
6	2	2	1.13	56.5
	3	1	.22	22.0
	4	2	.97	48.5
	5	2	1.58	79.0
7	1	2	.74	37.0
	2	1	.48	48.0
	3	2	.67	33.3
	4	1	.48	47.5
	4	2	.48	23.8
8	4	2	1.01	50.3
	5	1	.16	16.0
9	2	2	.99	49.5
	3	2	1.56	78.0
	5	2	.95	47.3
10	2	2	1.21	60.5
	3	2	.58	29.0
	4	1	.50	49.5
	5	2	1.66	82.8

Initial factor analysis of the 36 SJT items converged in 33 rotations and resulted in 15 factors explaining 64.1 % of the variance in SJT scores. Correlations between items ranged from –.19 to .40, with an average of .04. Kaiser’s criterion was not met as communalities were less than 0.7 [50]. Therefore a scree plot was used to retain 10 factors for the final analysis, explaining 48.9 % of the variance in candidate scores. The 10 retained factors displayed no consistent patterns.

Table 4 shows Pearson’s correlation coefficients for pre-admissions variables, SJT score and HEXACO-PI dimensions as well as descriptive statistics and Cronbach’s alpha reliabilities where available. Raw correlations were corrected for attenuation resulting from unreliability, which provides an estimate of the correlation between measures should this form of measurement error be reduced [52].

**Table 4:** Correlations between pre-admissions measures and the HEXACO personality inventory, descriptive statistics and Cronbach’s alpha reliabilities; coefficients within brackets are corrected for attenuation

	Measure	1	2	3	4	5	6	7	8	9	10
1	Academic Score	-									
2	UKCAT Score	.09	-								
3	MMI Total	.11	–.03	-							
4	SJT Score	–.19**	–.11	.32** (.47)	-						
5	Honesty - Humility	–.13	–.11	.03 (.04)	.36** (.53)	-					
6	Emotionality	.01	.04	.24** (.34)	.10 (.14)	–.04 (-.05)	-				
7	Extraversion	–.01	–.07	.30** (.41)	.29** (.40)	.12 (.15)	-.03 (-.04)	-			
8	Agreeableness	–.05	–.19**	-.05 (–.07)	.16* (.22)	.28** (.36)	–.14 (–.18)	.16* (.19)	-		
9	Conscientiousness	–.05	–.14	.18* (.25)	.36** (.50)	.26** (.34)	.13 (.17)	.36** (.44)	.19** (.23)	-	
10	Openness	.04	.15*	.10 (.14)	.06 (.08)	.20** (.26)	.07 (.09)	.08 (.10)	–.06 (–.07)	.02 (.03)	-
	*Mean*	5.0	2752.4	104.8	33.6	62.0	48.8	62.1	56.3	62.9	55.8
	*SD*	0.4	167.6	14.1	7.4	6.8	7.0	7.7	7.9	7.3	8.3
	*Range*	4–6	2310–3310	63–138	15–53	42–77	30–67	42–79	33–80	39–78	35–78
	*Alpha*	-	-	.71	.64	.73	.77	.83	.82	.80	.79

*p < .05; **p < .01

Statistically significant correlations between SJT score and personality dimensions ranged from .16 to .36 and .22 to .53 after correction for attenuation. By order of magnitude these relationships pertained to the honesty-humility (integrity), conscientiousness, extraversion and agreeableness dimensions. Statistically significant correlations between MMI score and personality dimensions ranged from .18 - .30 and .25 - .41 after correction for attenuation. By order of magnitude these relationships pertained to the extraversion, emotionality and conscientiousness dimensions.

Statistically significant correlations were also observed between SJT and MMI scores (.32; .47 disattenuated) as well as academic scores (-.19). The relationship between SJT and UKCAT scores was not statistically significant.

One hundred and ninety-six (99.0 %) applicants completed the acceptability questionnaire. Table 2 shows the average ratings given by candidates to the five questionnaire items. Mean scores ranged from 3.4-4.4. Cronbach’s alpha reliability of the acceptability questionnaire was 0.77.

## Discussion

This study aimed to explore the construct and acceptability of a new measure of integrity using SJT methodology for potential use in medical school admissions. Whilst one author hypothesised that SJT factor analytic results would indicate context specificity by showing distinct clusters of scenario-specific items [26], no clearly discernible patterns were found. This is consistent with the findings of McDaniel and Whetzel whose review found no evidence of a consistent and interpretable SJT factor structure, which they argued was due to the multidimensional nature of typical SJTs [53].

Multidimensionality has been argued to be inherent to SJT methodology as tests are often developed through critical incidents, which almost always demand multiple knowledge, skills abilities or traits for successful resolution [54]. This may therefore explain the current SJT’s associations with multiple personality dimensions, as the determination of an integrity-related action’s appropriateness may also reflect other personality traits. For example Scenario 7’s theft-related theme, while clearly conceptually associated with integrity, can also be linked to agreeableness and conscientiousness as low scorers on these dimension have been associated with criminal acts [55]. Further research should assess the relationships between specific integrity-related SJT items and personality dimensions.

The moderate correlations observed between SJT scores and the Honesty-Humility dimension of the HEXACO were larger than those reported by both De Meijer et al and Becker et al, and were not associated with cognitive ability as measured by the UKCAT [23, 24]. These correlations are encouraging as the SJT content was not derived from HEXACO items and are also distinct with respect to question style, response format and test construction methodology. This suggests a significant organic overlap between the integrity-related SJT scenarios in a medical school setting and integrity as a personality trait. Promising evidence is therefore provided for the successful development of an integrity-based SJT.

The small to large disattenuated correlations observed between SJT score, extraversion, agreeableness and conscientiousness are encouraging as these personality dimensions have been shown to be correlates of medical school outcomes [56–58]. Extraversion has been linked to medical school GPA in both pre-clinical and clinical years- suggesting it may be predictive of the collaboration and interpersonal skills needed as a medical student in academic studies as well as the clinical environment [56]. Agreeableness-related personality traits have also been shown to be predictors of clinical performance in both postgraduate and undergraduate samples [59, 60]. There is also strong evidence that conscientiousness is associated with academic performance throughout medical school [56, 57, 61], with validities increasing with each progressing year. Furthermore a general consensus exists in research settings outside of medicine that conscientiousness is a valid predictor of integrity-related outcomes including theft, disciplinary issues and rule-breaking [62].

Moderate associations were observed between SJT and MMI score, which is to be expected given that the MMI is designed to measure non-cognitive characteristics. This is particularly encouraging as MMIs have been shown to be predictive of performance at medical school [9–11]. While these correlations are smaller than those reported by Dore et al. [18], it should be noted that their SJT measured a broader range of constructs which overlap more with those assessed in the MMI.

The overall associations with personality and the MMI suggest that assessing the predictive validity of an integrity focused SJT appears worthwhile. As a reliable and realtively cheaply applied test (online or written), an SJT may be used as a pre - interview screening tool or could partially replace MMI content, thereby enabling a greater proportion of applicants to be assessed with fewer resources. This is particularly relevant in undergraduate selection to medical school as the UKCAT now includes an SJT component which also aims to measure personal qualities among prospective medical students [22]. The results of this study therefore lend some support to the UKCAT’s implementation of SJTs for selection.

This study also considered the relationship between SJT score and cognitive ability, measured using the UKCAT aptitude test and prior academic achievement. The SJT score was not significantly associated with UKCAT score, supporting the use of the SJT as a non-cognitive assessment with little or no redundancy with cognitive ability.

It is notable that SJT score correlated negatively with academic attainment. Whilst evidence exists that a high level of academic attainment is a justified prerequisite for medical study [1–3] it is intriguing that this may run counter to personal integrity. These findings may be supported by Powis & Bristow who found an association between high academic and poor structured interview scores, suggesting that selection based heavily on academic attainment may weigh against the non-academic attributes we seek [63]. Further exploration of this finding is required.

Gender differences were also observed among SJT scores, and while not desirable, this is consistent with previous findings related to gender differences in MMIs [9], other SJTs [16, 28] and other medical school and postgraduate exams. Further research should investigate whether differential construct or predictive validities are present across genders. It is also possible that valid and reliable scores for meaningful core personal attributes select a higher proportion of females and this should be investigated further.

Acceptability evidence was in line with those of other authors evaluating the use of SJTs for selection, with most candidates agreeing that the SJT was realistic and relevant to the role of medical student [16, 20–22, 58, 64]. Notably, candidates did not agree that the SJT was obviously related to the role of medical student from an outsider’s perspective (Question 3) while agreeing that they themselves saw the relationship between the SJT and the role (Question 1). As test perceptions can affect people’s attitudes [43, 65] further research should investigate whether specific scenario types are associated with low acceptability, and whether this in turn is associated with SJT performance.

One limitation of this study is its inability to explore the representativeness of the SJT group to the wider applicant pool as participants were selected to interview based on high UKCAT and academic scores. Further research should investigate the psychometric characteristics of an SJT to the population of applicants or a representative sample.

The results demonstrate the development of an integrity-based SJT for undergraduate selection into UK medical training with acceptable reliability, construct validity and face validity. Future research should focus on seeking further evidence of construct validity with other measures of integrity as well as evidence of predictive validity for the SJT developed in this study. Future studies should also explore the versatility of SJTs to select for other non-academic measures, potentially leading to the introduction of a comprehensive suite of selection tools combining MMIs and SJTs to target specific personal qualities. As such, participants who completed the SJT described here should be followed-up both during medical school and their further clinical training.

## Conclusions

Initial findings are encouraging regarding the psychometric robustness of an integrity-based SJT for medical school selection with acceptable reliability, construct validity and face validity. Results suggest that carefully-designed SJTs may augment more costly MMIs.

## Abbreviations

SJT: Situational judgement test
MMI: Multiple mini-interview
OSCE: Objective structured clinical examination
UCAS: Universities and colleges admissions service
UKCAT: United Kingdom Clinical Aptitude Test
